# Detection of Outliers Due to Participants’ Non-Adherence to Protocol in a Longitudinal Study of Cognitive Decline

**DOI:** 10.1371/journal.pone.0132110

**Published:** 2015-07-10

**Authors:** Aline Dugravot, Severine Sabia, Martin J. Shipley, Catherine Welch, Mika Kivimaki, Archana Singh-Manoux

**Affiliations:** 1 INSERM, U1018, Centre for Research in Epidemiology and Population Health, Villejuif, France; 2 Université Paris-Sud 11, 82 rue du Général Leclerc, Le Kremlin-Bicêtre Cedex, France; 3 Department of Epidemiology and Public Health, University College London, London, United Kingdom; 4 Université de Versailles Saint-Quentin, Versailles, France; Cardiff University, UNITED KINGDOM

## Abstract

**Background:**

Participants’ non adherence to protocol affects data quality. In longitudinal studies, this leads to outliers that can be present at the level of the population or the individual. The purpose of the present study is to elaborate a method for detection of outliers in a study of cognitive ageing.

**Methods:**

In the Whitehall II study, data on a cognitive test battery have been collected in 1997-99, 2002-04, 2007-09 and 2012-13. Outliers at the 2012-13 wave were identified using a 4-step procedure: (1) identify cognitive tests with potential non-adherence to protocol, (2) choose a prediction model between a simple model with socio-demographic covariates and one that also includes health behaviours and health measures, (3) define an outlier using a studentized residual, and (4) study the impact of exclusion of outliers by estimating the effect of age and diabetes on cognitive decline.

**Results:**

5516 participants provided cognitive data in 2012-13. Comparisons of rates of annual decline over the first three and all four waves of data suggested outliers in three of the 5 tests. Mean residuals for the 2012-13 wave were larger for the basic compared to the more complex prediction model (all p<0.001), leading us to use the latter for the identification of outliers. Residuals greater than two standard deviation of residuals identified approximately 7% of observations as being outliers. Removal of these observations from the analyses showed that both age and diabetes had associations with cognitive decline similar to that observed with the first three waves of data; these associations were weaker or absent in non-cleaned data.

**Conclusions:**

Identification of outliers is important as they obscure the effects of known risk factor and introduce bias in the estimates of cognitive decline. We showed that an informed approach, using the range of data collected in a longitudinal study, may be able to identify outliers.

## Introduction

Non adherence to protocol is a source of bias in studies, irrespective of the design or nature of the study. A recent review concluded that a majority of RCTs, the gold-standard in research studies, are subject to some form of non-adherence to treatment protocol.[1] The standard method of analysis, intention to treat ignores information on non-adherence. It has been suggested that further analyses of such trials are needed so that compliance and withdrawal can be taken into account by censoring the subject at the time of deviation from protocol.[1,2] In observational studies, non-adherence to protocol may give rise to outliers, defined simply as observations that are extreme or unusual with respect to rest of the data.[3] Outliers could result from variability in the attribute being assessed or measurement error. In the first case, no further action is necessary apart from ensuring that the statistical method for analysis is robust to outliers.[4,5] Error in a data point could stem from mistakes during the measurement or transcription of data, or due to the issues of social desirability, misreporting, or non-adherence to protocol. In such cases, outliers are usually removed, most often using the observed distribution.[6]

The identification of outliers in longitudinal data is not straightforward as they may be present both at the level of the population and the individual.[7] Thus, an individual can be an outlier with respect to the rest of the sample or with respect to their own previous or subsequent measurements on an attribute. In the first case, the Gaussian distribution has long been used to identify observations that could be erroneous.[6] While this procedure reduces sample size, it ensures that the estimations of exposure-outcome associations obtained from the data are less biased than those obtained prior to data cleaning. The identification of outliers in longitudinal data is more complex, particularly if the aim of the study is to examine changes over time in the attribute being assessed.

The objective of this paper is to develop procedures to detect outliers in an investigation of cognitive decline on 5516 persons participating in the Whitehall II study. Four waves of data have been collected over the past 15 years and the current exercise was prompted by observation of non-adherence to protocol by some participants at the fourth wave of data collection. Data collection in the Whitehall II study is undertaken in a clinical setting for most participants and at home for those unable or unwilling to travel to the clinic. Part of the cognitive test battery is administered in a group setting, with instructions administered via headphones and this is where non-adherence to protocol was observed during data collection. In order to identify outliers we used residuals, the commonly used diagnostic method to identify observations that are different from expected values calculated using a prediction model.[8] We used two types of prediction models, a simple model based on socio-demographic factors and previous data on the cognitive test in question and a second model which additionally included a wide range of behavioural and health factors shown to be associated with cognition in the literature and our previous analyses. We also compared outliers and others, including analysis of the impact of exclusion of outliers on associations with known risk factors, ageing and diabetes, with cognitive decline.

## Materials and Methods

### Study Population

The Whitehall II study is an ongoing cohort study of men and women, originally employed by the British civil service. The study was setup to study social determinants of health, cardiovascular disease and diabetes in particular. The target population was all London-based office staff, aged 35–55 years. A total of 10,308 persons (6895 men and 3413 women), response 73%, were recruited to the study over the years 1985 to 1988.[9] Although all participants in the study are white-collar workers, a wide range of occupations is represented with a salary difference of over 10-fold between the top and bottom of the socioeconomic hierarchy. All participants responded to a questionnaire and underwent a uniform, structured clinical evaluation, consisting of measures of anthropometry, cardiovascular and metabolic risk factors and disease. Since the baseline medical examination, follow-up examinations have taken place approximately every 5 years: 1991–93 (n = 8815); 1997–99 (n = 7870); 2003–04 (n = 6967), 2007–09 (n = 6761) and 2012–13 (n = 6318). The clinical assessment takes two years to complete and is organized in Central London, with travel arrangements for the participants made by study staff. Starting from the 2003–2004 assessments participants who were unable or unwilling to travel to London have been offered a clinical assessment at home; 11.4% (2003–04), 13.4% (2007–09) and 17.7% (2012–13) have taken this option.

All participants gave written informed consent. Participant consent and research ethics approvals (University College London (UCL) ethics committee) were renewed at each contact; latest approved was by the Joint UCL/UCLH Committee on the Ethics of Human Research (Committee Alpha), reference number 85/0938.

#### Cognitive function: response in a single booklet

The cognitive test battery was introduced to the study in 1997–99. The tests were chosen to provide a comprehensive assessment of cognitive function and be appropriate for this population composed of individuals younger than in most studies on cognitive ageing. The core battery has been administered four times over 15 years (in 1997–1999, 2003–2004, 2007–2009 and 2012–13). These tests have good test-retest reliability (range 0.60–0.89) in 556 participants who were invited back for retesting within three months of having taken the tests. The instructions were recorded and administered via headphones, both at the clinic and at home, and responses recorded in a booklet. The difference between the two settings was that at home the tests were administered in a one to one setting whereas at the clinic they were administered to up to five participants at the same time. These tests are described below in the sequence in which they were administered during the assessment.

Short term verbal memory was assessed with a 20 word free recall test. Participants were presented orally a list of 20 one or two syllable words at two second intervals and were then asked to recall in writing as many of the words in any order within two minutes.

Reasoning was assessed using the Alice Heim 4-I[10] (AH4-I), composed of a series of 65 verbal and mathematical reasoning items of increasing difficulty. It tests inductive reasoning, measuring the ability to identify patterns and infer principles and rules. Participants had 10 minutes to do this section.

Vocabulary was assessed with the Mill Hill vocabulary test,[11] used in its multiple choice format, consisting of a list of 33 stimulus words ordered by increasing difficulty and six response choices. Participants had ten minutes to complete this section.

Verbal fluency was assessed using tests of phonemic and semantic fluency.[12] Participants were asked to recall in writing as many words beginning with “S” (phonemic fluency) and as many animal names (semantic fluency) as they could. One minute was allowed for each test; the observed range on these tests was 0–35.

#### Cognitive function: one to one administration

The Mini-Mental State Examination, a brief 30-point measure of global cognitive function [13], was administered to participants 60 years and older in 1997–99 and to all participants at subsequent waves of data collection. In 2012–13, Parts A and B of the Trail Making Test (TMT) were added to the cognitive battery as a measure of visual speed and executive function.[14] The MMSE and TMT data were not used in the detection of outliers as they were not available longitudinally but were used to compare outliers to others.

#### Additional measures used in prediction models

Besides prior data on cognitive tests, we also used a range of covariates in the prediction model. The choice of these covariates was based on evidence showing them to be associated with cognition and their availability in the study.

Socio-demographic factors[15–17] included year of birth (1930–34; 1935–39; 1940–44; 1945–49; 1950–52), sex, age, marital status (married/cohabiting, single, divorced and widowed), ethnicity (white and non-white), socioeconomic status (SES), assessed via occupational position (6 levels)[9], and education (elementary, lower secondary, higher secondary, first university degree and higher).

Health behaviours[16,18,19] considered were smoking status (never, current, or ex-smoker), alcohol intake (units of alcohol consumed in the past 7 days), physical activity (sum of the product of the intensity (metabolic equivalent (MET)[20]) and weekly duration of all moderate and vigorous activity, MET-hours/week), and consumption of fruit and vegetables (assessed using the question, “How often do you eat fresh fruit or vegetables?”, responses were on an eight-point scale, ranging from “seldom or never” to “two or more times a day”).

Health measures included cardiovascular risk factors[16,18,21] and diseases,[22,23] self-rated general health,[24] mental disorder symptoms,[16,18,25] and use of related medications. Cardiovascular risk factors considered were as follows: body mass index calculated as the ratio of weight (kilograms) to height (metres squared) and categorised using the World Health Organization classification (underweight, <18.5 kg/m^2^; standard weight, 18.5–24.9 kg/m^2^; overweight, 25–29.9 kg/m^2^; obese, 30–34.9 kg/m^2^ and severely obese ≥35 kg/m^2^); resting heart rate measured via electrocardiogram with participants in the supine position and categorized as < 60, 60–80, and > 80 beats/minute; systolic and diastolic blood pressure measured using the Hawksley random-zero sphygmomanometer, average of two consecutive measures with the participant sitting after a 5-minute rest; fasting serum cholesterol measured within 72 hours in serum stored at 4°C using enzymatic colorimetric methods. Diabetes was defined by a fasting glucose ≥7.0 mmol/L or reported doctor diagnosed diabetes, or use of diabetes medication.[26] CHD prevalence was based on clinically verified events and included myocardial infarction and definite angina.[27] Stroke cases were ascertained from participants’ general practitioners, information extracted from hospital medical records by study nurses, or data from the National Health Service (NHS) Hospital Episode Statistics database obtained after linking the participants’ unique NHS identification numbers to this national database.[28] Use of central nervous system medication and cardiovascular disease medication were also included. Measures of *general* health included the physical and mental components scores of the SF-36 questionnaire[29] and response to the following question ‘In general, would you say your health is:’ with a 5-point Likert scale from “Excellent” to “poor”. Common mental disorder symptoms were assessed using the 30-item General Health Questionnaire.[30]

The site (home or clinic) of the examination and number of times participant participated in cognitive testing over the follow-up the were also included in the model to take into account difference in protocol and practice effects,[31] respectively.

### Statistical methods

The analysis was undertaken in a sequential manner, described below.

#### Step 1. Identify cognitive tests with potential non-adherence to protocol

Rigorous protocol checks during test administration revealed potential non-adherence in the ten minutes allocated to the vocabulary test, part of the five tests administered in a group setting in the clinic. Some participants were found to be non-compliant with the instructions and were using part of the ten minutes to complete reasoning or fluency sections, all three being timed tests. We hypothesized that non-adherence would lead individual cognitive trajectories to differ when including the last wave of data compared to previous waves. In order to identify the specific tests that were affected we compared annual change in test scores as estimated from annual change between 1997–1999 and 2007–09 using data from the first three assessments and that between 1997–1999 and 2012–13 using data from all four assessments. This exercise was undertaken by first calculating the annual change over the two follow-ups using individual ordinary least squares (OLS) slope from a linear regression and then comparing them with Student’s test for paired data. In order to be included in these analyses, participants needed to have at least two data points scores between 1997–99 and 2007–09, and data at the 2012–13 wave. In order to ensure that outliers were confined to persons seen at the clinic, these comparisons were undertaken in analyses stratified by site of test administration.

#### Step 2. Choice of prediction model

The purpose of this exercise was to predict, for each individual their cognitive test score in 2012–2013. It only concerned tests where there was evidence, based on step 1, of unusual patterns of change in test scores over time. The prediction models were based on linear mixed models using previous data on the test in question (1997/99, 2002/04, 2007/09) and included random terms for intercept and slope to take into account the fact that repeated measures on the same individual are correlated with each other.[32] We used two types of prediction models, a simple model which included age (centered at age 60 years), age squared, year of birth, sex, marital status, ethnicity, occupational position, education, and the interaction terms of these covariates with age and age squared. The second model included a wider range of covariates in order to improve the prediction. In addition to the variables in the simple model, it included health behaviours, health measures, the site of examination, the number of times a participant undertook the test in question, scores on cognitive tests deemed to be free of bias in step 1, and the interaction terms of each covariate with age and age squared. The interaction terms of the number of times the participant undertook the cognitive test with sex and SES were also included in the model. Covariates were included in the prediction model as time-dependent except when not appropriate (e.g. ethnicity). When data on covariates were missing they were imputed using multiple imputations, chained equations method, based on all available data on cognition and covariates. In both approaches, the predicted value for each participant was based on the fixed-portion of the linear predictor plus predicted random effects.

#### Step 3. Define an outlier

An outlier was defined simply as an observation with a large residual, i.e. a large discrepancy between the observed and predicted value at the 2012–2013 assessment. Absolute thresholds are difficult to establish making standardized residuals (residual divided by its standard deviation, SD) a useful method that allows similar thresholds to be used across tests with the convention being to use 2 SD to identify unusual observations. Our interest was only in scores that were more than 2 SD above the mean in order to detect unusual improvement in scores (a large deterioration in score is possible due to neurological disorder). The SD to be used in defining the outliers may be defined in a number of ways. One option would have been to use the 2012–13 wave of data, but given the influence that the outliers would have on the SD, we instead calculated the SD of the residuals from the prediction model based on the first three waves of data.

#### Step 4. Study impact of exclusion of outliers

We first compared annual cognitive change based on the first three waves of data and on all four waves for the given cognitive test before and after exclusion of outliers at the fourth wave. In addition, mixed models were used to examine the association of age and diabetes, both known risk factors cognitive decline, assessed in 1997–99, with cognitive decline over 15 years (1997–99 to 2012–13), in both samples including and excluding outliers.

All analyses were performed using Stata SE version 12 for Windows (StataCorp. 2011).

## Results

**Fig 1:**
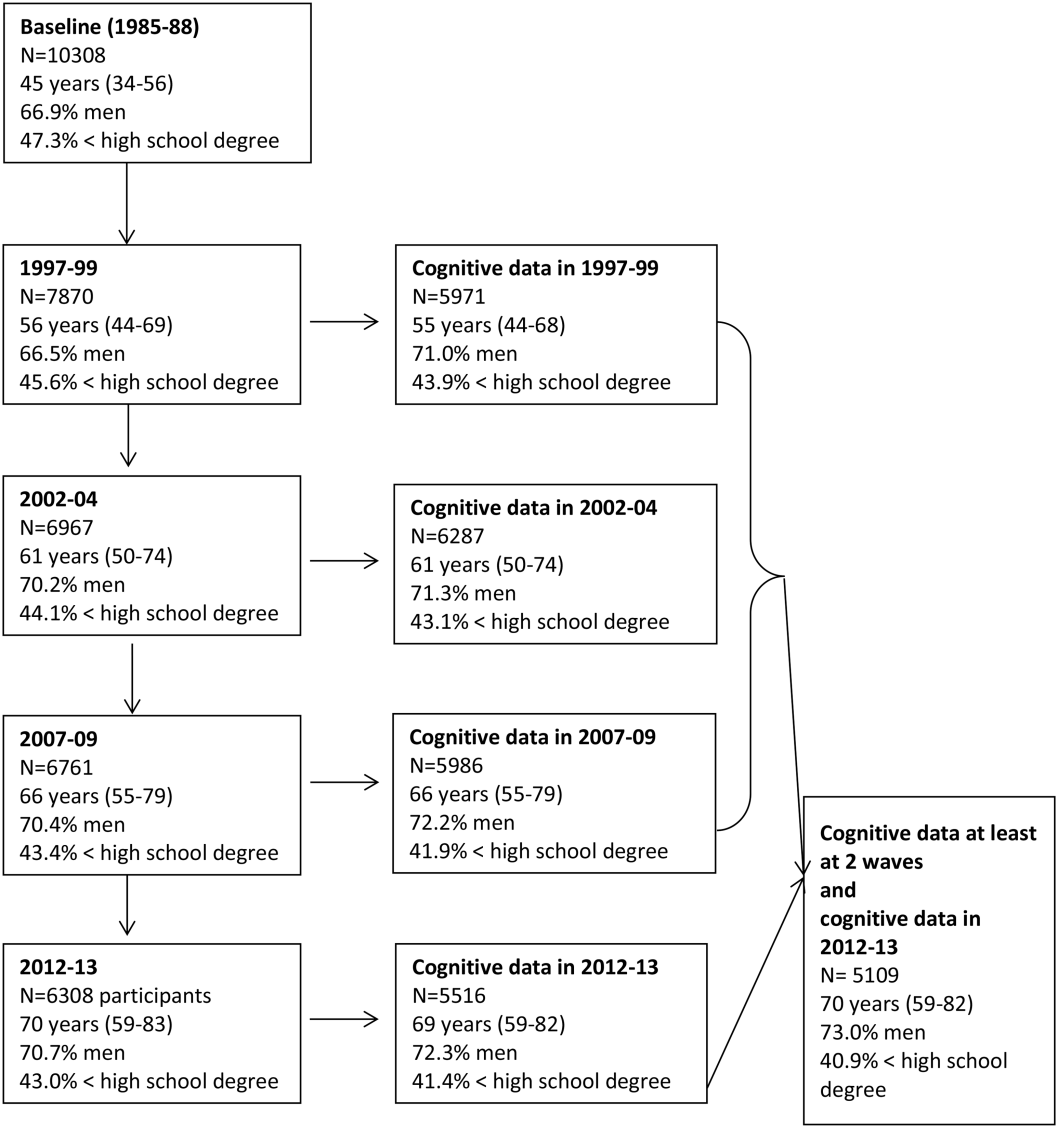
Sample selection, Whitehall II Study.

Fig 1 shows a flow chart of the sample selection. Of the 10308 participants at study inception in 1985–88, 6308 (61%) provided data in 2012–13. They were on average 70 years old (range = 59–83), 70.7% were men and 43.0% had an educational level less than a high school diploma. Overall, 5109 participants had cognitive data at least at two waves prior to, and in 2012–13, for at least one of the five cognitive tests.

### Step 1: Identify cognitive tests with potential non-adherence to protocol

Table 1, top panel, shows that compared with annual change estimated using data from the first 3 waves, estimates using all 4 waves show similar decline in memory (p = 0.88) but slower declines in reasoning (-0.28 vs -0.24; p<0.001), phonemic fluency (-0.16 vs -0.13; p<0.001), and semantic fluency (-0.13 vs -0.12; p = 0.02). This difference was evident in participants seen at the clinic (mid panel) but not in those seen at home (bottom panel).

**Table 1:** Comparison of annual change between 1997–99 and 2007–09 with that between 1997–99 and 2012–13.

	N	1997–99 to 2007–09 M (SD)	1997–99 to 2012–13 M (SD)	p-value
**ALL PARTICIPANTS IN 2012–2013**				
Memory	5067	-0.07 (0.27)	-0.07 (0.18)	0.875
Reasoning	5104	-0.28 (0.65)	-0.24 (0.45)	<0.001
Phonemic fluency	5104	-0.16 (0.42)	-0.13 (0.27)	<0.001
Semantic fluency	5108	-0.13 (0.37)	-0.12 (0.24)	0.02
Vocabulary	5109	0.02 (0.26)	0.01 (0.18)	0.01
**ASSESSMENT AT CLINIC IN 2012–13**				
Memory	4279	-0.07 (0.26)	-0.07 (0.18)	0.29
Reasoning	4300	-0.28 (0.63)	-0.22 (0.44)	<0.001
Phonemic fluency	4300	-0.16 (0.40)	-0.12 (0.26)	<0.001
Semantic fluency	4304	-0.12 (0.36)	-0.11 (0.24)	0.02
Vocabulary	4304	0.02 (0.24)	0.01 (0.17)	0.17
**ASSESSMENT AT HOME IN 2012–13**				
Memory	788	-0.09 (0.33)	-0.11 (0.20)	0.10
Reasoning (AH4-I)	804	-0.29 (0.73)	-0.31 (0.50)	0.16
Phonemic fluency	804	-0.15 (0.50)	-0.15 (0.31)	0.64
Semantic fluency	804	-0.15 (0.42)	-0.14 (0.27)	0.75
Vocabulary	805	0.01 (0.34)	-0.02 (0.23)	0.01

Abbreviations: M mean, SD standard deviation.

### Step 2: Prediction models and outliers identification

Further analysis focused only on reasoning and the two fluency tests. Mean residuals (table 2) for the 2012–13 wave (observed minus predicted values) were larger for the basic compared to the more complex prediction model for reasoning (Mean(M) = 1.68, Standard Deviation (SD) = 4.69 vs. M = 0.44, SD = 4.75; p<0.001), phonemic fluency (M = 0.54, SD = 2.96 vs. M = 0.22, SD = 2.86; p<0.001), and semantic fluency (M = 0.34, SD = 2.64 vs. M = 0.09, SD = 2.58; p<0.001). The larger residuals in the simple model suggest that it fits the data less well than the more complex model.

**Table 2:** Comparison of residuals: from 1997–99 to 2007–09 compared with 2012–13.

		Across first 3 waves (1997–99 to 2007–09)	At fourth wave (2012–13)	Threshold used to define outliers*
	Range	M (SD)	M (SD)	
**Simple prediction model**				
Reasoning (AH4-I)	0–65	0.00 (2.96)	1.68 (4.69)	5.92
Phonemic fluency	0–35	0.00 (1.92)	0.54 (2.93)	3.84
Semantic fluency	0–35	0.00 (1.71)	0.34 (2.64)	3.42
**Widely adjusted prediction model**				
Reasoning (AH4-I)	0–65	0.00 (2.98)	0.44 (4.75)	5.96
Phonemic fluency	0–35	0.00 (1.94)	0.22 (2.86)	3.88
Semantic fluency	0–35	0.00 (1.77)	0.09 (2.58)	3.54

*Represents 2SD of the residual from the prediction model across first 3 waves.

Outliers were identified only among participants seen at the clinic, with residual values (observed minus predicted) that were higher than two standard deviations from the prediction models based on the first three waves of data collection (see thresholds to define outliers in Table 2). Using these thresholds identified 743 outliers for reasoning using the simple prediction model and 434 outliers with the complex model. For phonemic fluency the outlier numbers were 493 and 377 in the simple and complex model, the corresponding numbers were 495 vs 370 for semantic fluency. Given the better fit of the complex model and the smaller numbers of outliers identified by this model it was chosen as the preferred method.

### Step 3: Outlier characteristics

Outliers were not different from others in terms of age, sex, and education in a systematic manner across the three cognitive tests (Table 3). However, their cognitive test scores in 2012–13 were higher on all tests with outliers (all p<0.001) such that the scores on these tests over the four waves showed outliers to increase their cognitive scores, whereas other participants experienced cognitive decline. However, restricting analyses to the first three waves of data showed that outliers in 2012–13 had experienced greater annual decline over the first three waves of data collection compared to others (Table 3). On the other tests at the 2012–13 assessment, the differences on MMSE, memory and vocabulary scores were not systematic although outliers had quicker speed of completion of the TMT A & B (all p<0.01).

**Table 3:** Characteristics of outliers compared to others assessed at the clinic in 2012–13.

	Reasoning (N = 5516)			Phonemic fluency (N = 5513)			Semantic fluency (N = 5516)		
2012–13 characteristics	Outliers	Others	age adjusted p value*	Outliers	Others	age adjusted p value*	Outliers	Others	age adjusted p value*
N (%)	434 (7.87)	5082 (92.13)		377 (6.84)	5136 (93.16)		370 (6.71)	5146 (93.29)	
Age (years)	70.43 (5.58)	69.41 (5.79)	<0.001	68.63 (5.53)	69.56 (5.79)	0.003	69.15 (5.77)	69.52 (5.78)	0.23
Men, N (%)	298 (68.66)	3692 (72.65)	0.08	269 (71.35)	3720 (72.43)	0.62	286 (77.30)	3704 (71.98)	0.03
Education, N (%)									
Lower secondary	199 (45.85)	2083 (40.99)		125 (33.16)	2155 (41.96)		125 (33.78)	2157 (41.92)	
Secondary school	116 (26.73)	1387 (27.29)	0.31	113 (29.97)	1390 (27.06)	0.02	104 (28.11)	1399 (27.19)	0.01
University	119 (27.42)	1612 (31.72)		136 (36.87)	1591 (30.98)		141 (38.11)	1590 (30.90)	
**Test score in 2012–13**	48.45 (10.25)	42.85 (11.34)	<0.001	21.44 (4.20)	14.70 (3.94)	<0.001	20.33 (3.17)	14.55 (3.73)	<0.001
**Annual cognitive change**									
**1997–99 to 2007–09**	-0.43 (0.77)	-0.27 (0.63)	<0.001	-0.24 (0.52)	-0.15 (0.41)	<0.001	-0.21 (0.43)	-0.12 (0.36)	<0.001
**1997–99 to 2012–13**	0.25 (0.60)	-0.26 (0.48)	<0.001	0.21 (0.35)	-0.15 (0.29)	<0.001	0.15 (0.31)	-0.14 (0.27)	<0.001
**Score on cognitive tests not part of outlier detection (2012–13)**									
Memory	5.55 (2.19)	6.05(2.39)	0.002	6.27 (2.47)	5.99 (2.37)	0.21	6.29 (2.36)	5.99 (2.38)	0.04
Vocabulary	24.22 (6.20)	25.26 (4.59)	<0.001	25.38 (50.4)	25.17 (4.71)	0.59	26.05 (4.29)	25.12 (4.77)	<0.001
MMSE	28.14 (1.60)	28.35 (1.58)	0.08	28.35 (1.37)	28.33 (1.59)	0.65	28.45 (1.36)	28.32 (1.59)	0.26
Trail Making Test A	37.87 (14.99)	39.04 (18.17)	0.01	34.78 (11.10)	39.25 (18.27)	<0.001	34.30 (11.54)	39.28 (18.27)	<0.001
Trail Making Test B	81.42 (37.41)	85.22 (39.02)	0.001	76.26 (33.21)	85.54 (39.22)	<0.001	73.15 (27.55)	85.77 (39.46)	<0.001

*For difference between outliers and non-outliers

Figures are means (SD) unless stated otherwise.

### Step 4: Impact of exclusion of outliers

Prior to exclusion of outliers the annual change in reasoning, phonemic and semantic fluency was larger using data from the first three waves compared to that using all four waves (Table 4). After exclusion, the rates were similar for reasoning (p = 0.46) and phonemic fluency (p = 0.96) and the decline stronger using all 4 waves instead of the first 3 waves for semantic fluency (-0.13 vs -0.12, p = 0.001).

**Table 4:** Mean annual change over the first 3 waves (1997/99 to 2007/09) and over all 4 waves (1997/99 to 2012/13) before and after exclusion of outliers.

	BEFORE EXCLUSION OF OUTLIERS				AFTER EXCLUSION OF OUTLIERS			
	N	1997–99 to 2007–09	1997–99 to 2012–13		N	1997–99 to 2007–09	1997–99 to 2012–13	
		M (SD)	M (SD)	p-value		M (SD)	M (SD)	p-value
**Reasoning (AH4-I)**	5104	-0.28 (0.65)	-0.24 (0.45)	<0.001	4725	-0.27 (0.63)	-0.27 (0.44)	0.46
**Phonemic fluency**	5104	-0.16 (0.42)	-0.13 (0.27)	<0.001	4757	-0.15 (0.41)	-0.15 (0.25)	0.96
**Semantic fluency**	5108	-0.13 (0.37)	-0.12 (0.24)	0.02	4763	-0.12 (0.36)	-0.13 (0.24)	0.001

Abbreviations: M mean, SD standard deviation.

The effect of 10 years of ageing, adjusted for year of birth, sex and education, was a -2.76 (95%CI = -2.87,-2.65) before and -3.11 (95%CI = -3.22, -3.00) after exclusion of outliers for reasoning (data not tabulated). Corresponding figures were -1.33 (95%CI = -1.39, -1.26) and -1.54 (95%CI = -1.61, -1.48) for phonemic fluency and -1.20 (95%CI = -1.26, -1.14) and –1.37 (95%CI = -1.43, -1.31) for semantic fluency. Exclusion of outliers led to an increase in the effect of ageing between 12.7% and 15.8%.

Table 5 presents the association of diabetes with 10-year cognitive change before and after exclusion of outliers. For reasoning and semantic fluency, associations were similar in both samples. The association between diabetes and decline in phonemic fluency became evident after exclusion of outliers.

**Table 5:** Estimated difference in cognitive decline over 10 years, as a function of diabetes status in 1997/99.

	Before exclusion	After exclusion
	Beta^†^ (95% CI)	Beta^†^ (95% CI)
**Reasoning (AH4-I)**		
	Nobs = 20847 N = 6269	Nobs = 20482 N = 6269
Normoglycaemia	*Ref (-2*.*80* ^‡^ *(-2*.*91*, *-2*.*69))*	*Ref (-3*.*14* ^‡^ *(-3*.*25*, *-3*.*03))*
Diabetes	-0.79 (-1.38, -0.19)*	-0.84 (-1.45, -0.24)*
**Phonemic fluency**		
	Nobs = 20881 N = 6281	Nobs = 20556 N = 6281
Normoglycaemia	*Ref (-1*.*33* ^‡^ *(-1*.*39*, *-1*.*26))*	*Ref (-1*.*53* ^‡^ *(-1*.*60*, *-1*.*47))*
Diabetes	-0.29 (-0.62, 0.05)	-0.46 (-0.79, -0.13)*
**Semantic fluency**		
	Nobs = 20882 N = 6282	Nobs = 20561 N = 6282
Normoglycaemia	*Ref (-1*.*20* ^‡^ *(-1*.*26*, *-1*.*14))*	*Ref (-1*.*37* ^‡^ *(-1*.*43*, *-1*.*31))*
Diabetes	-0.15 (-0.46, 0.15)	-0.16 (-0.46, 0.14)

*P<0.01.

^†^Models with age as time-scale and adjusted for year of birth, sex, education, and their interaction with age. Betas are for difference in cognitive change over 10 years.

^‡^ 10-year cognitive change in the reference group.

## Discussion

In response to observation of non-adherence to protocol at the fourth wave of cognitive data collection we undertook a search for outliers upon completion of data collection. This procedure identified approximately seven percent of data on three tests to be outliers. The outliers were not characterised by a specific socio-demographic profile, but we were able to show implausible “cognitive ageing” such that their cognitive scores improved over the follow-up, rather than declined as in the rest of the study sample, when data from the 2012–13 were included in the analyses. Analysis of the effects of age and diabetes, strong correlates of accelerated cognitive ageing, also show that not removing outliers from the analysis would result in underestimation of their effects.

The identification of outliers in studies is an inexact science. This is particularly the case in longitudinal data were there may be outliers at the within-person and between-person levels. Thus, an individual can be an outlier with respect to the rest of the population or an observation can be an outlier with respect to previous or subsequent measurements from an individual. In both cases, outliers generally serve to increase error variance, reduce the power of statistical tests, and bias estimates. Residuals are the most common diagnostic method to identify outliers, they are a gauge of how far a measurement is from the predicted value.[8] The earliest outlier test criterion suggested that observations whose residual exceeds in magnitude five times the probable error were likely to be outliers.[6] However, criteria based on simple multiples of the sample standard deviation for the detection of outliers is not optimal because of their interdependence: outliers lead to inflated standard deviations upon which outlier detection is based.[33] Outlier detection using fixed multiples is also inefficient as the number of measurements that are identified as outliers will be influenced by the size of the sample. One solution is the use of a studentized residual where the standard deviation is drawn from the population. Our solution is an adaptation of this strategy, the standard deviation used to define residuals was drawn from data at all waves prior to the one subject to outlier detection. Use of 2.5 instead of 2 standard deviations identified fewer data points (4.7%) as being outliers but did not correct the effects on cognitive decline (data not tabulated but available from authors).

Outliers can be real or erroneous; removing the former would be problematic as it would lead to the exclusion of genuine extreme values, resulting in underestimation of uncertainty present in the data. Longitudinal studies relate measurements made at several different times on a single individual, with the intention of studying change in processes such as growth or decline. The study of cognition in longitudinal studies is also known to be affected by biases such as retest or practice effects which arise out of growing familiarity with the tests and testing environment; decrease in stress and anxiety; recall effects; procedural learning; and actual learning over time. There are various statistical strategies for dealing with these effects, which have been well described by Ferrer and colleagues[33] and Rabbitt and colleagues.[31,34] These effects are important to consider as the effects of age can be cancelled out or obscured by hidden practice effects. Previous research suggests that practice effects decrease in magnitude over occasions;[33, 34] younger and higher ability persons have also been shown to gain more from practice.[34] The fact that we did not observe these patterns in our data at the 2012–13 wave of data collection suggests that our data are affected by outliers rather than a manifestation of practice effects. For example, education and age did not have a systematic association with being an outlier in our study. Thus, the most likely explanation for the elevated scores obtained by some participants is that the time allocated for the completion of the tests was not respected. The vocabulary test, which was placed in between the reasoning and the fluency tests, had ten minutes allocated to it and some of this time appears to have been used by some participants to “improve” performance on other tests. As this was the fourth wave of cognitive data collection, participants had become familiar both with the tests and the testing conditions. We believe it is familiarity with the testing procedure that has led to the improvement in scores. To confirm this, at the next wave of data collection, the vocabulary test will be removed, and the booklet separated into two in order to ensure that the time allocated to each test is strictly adhered to by the participants.

### Conclusions

The detection of outliers within the framework of longitudinal studies has received very little attention despite the plethora of studies on ageing. We used a prediction model to calculate residuals, which were then used to identify outliers. These outliers have been reset to be missing. This solution is particularly appealing in the context of a longitudinal study as it allows us to retain other data points from the individual in the analysis. As the study is ongoing we will be able to reassess our strategy when the next wave of data become available in 2017.
